# Oral microbiota in aging and diseases

**DOI:** 10.1093/lifemedi/lnae024

**Published:** 2024-06-28

**Authors:** Ya Ren, Mingxu Chen, Ziyang Wang, Jing-Dong J Han

**Affiliations:** Peking-Tsinghua Center for Life Sciences, Academy for Advanced Interdisciplinary Studies, Center for Quantitative Biology (CQB), Peking University, Beijing 100871, China; Peking-Tsinghua Center for Life Sciences, Academy for Advanced Interdisciplinary Studies, Center for Quantitative Biology (CQB), Peking University, Beijing 100871, China; CAS Key Laboratory of Computational Biology, CAS-MPG Partner Institute for Computational Biology, Shanghai Institute of Nutrition and Health, Chinese Academy of Sciences Center for Excellence in Molecular Cell Science, Collaborative Innovation Center for Genetics and Developmental Biology, Shanghai Institutes for Biological Sciences, Chinese Academy of Sciences, Shanghai 200031, China; Peking-Tsinghua Center for Life Sciences, Academy for Advanced Interdisciplinary Studies, Center for Quantitative Biology (CQB), Peking University, Beijing 100871, China; Peking-Tsinghua Center for Life Sciences, Academy for Advanced Interdisciplinary Studies, Center for Quantitative Biology (CQB), Peking University, Beijing 100871, China

**Keywords:** oral microbiome, aging, disease

## Abstract

Human microbiomes are microbial populations that form a symbiotic relationship with humans. There are up to 1000 species on the surface of human skin and mucosal system, among which gut microbiota attracts the most interest. As the beginning of the digestive tract, oral cavity is also an important microbial habitat in the human body which is the first line of defense against pathogens entering the body. Many studies have revealed that oral microbial dysbiosis could not only contribute to oral diseases but also whole-body systemic diseases and health status. Oral microorganisms can enter the gastrointestinal tract with saliva and food, or enter the blood circulation through mouth breakage, thus causing systemic inflammation and aging-related diseases including some causal links to Alzheimer’s disease. A series of changes take place in oral microbial composition during development, with different age stages marked by different dominant microbial species. Despite a lack of comprehensive studies on aging oral microbiota, through systemic inflammation, oral pathogenic microbes are likely to contribute inflammatory aging. As inflammaging is a key signature and one of the causes for accelerated aging, improving the structure of oral microbiome may be not only a new strategy for disease prevention and treatment, but also for aging intervention.

## Introduction

Aging is a process of time-dependent physiological functional decline in all aspects of a biological system which is the greatest risk factor for most human diseases. During aging, many physical disorders are characterized by immune function decline, metabolic disturbance, intestinal dysfunction due to gut flora changes, and so on [1]. In recent years, many studies have reported the contribution of gut microbial community to body health, including the effects on nutritional status, energy expenditure and immunity [2]. Notably, microbiome dysbiosis has been recognized as a hallmark of aging [1], and gut microbiome has been also found to play an important role in regulating health span and life span [3]. A healthy gut microbiome plays a key role in controlling metabolism, fighting infections and inflammation, preventing autoimmune diseases and cancer, and regulating the brain–gut axis [4].

Gut microbiota composition can change significantly with the occurrence of aging and senescence-related diseases, including a decrease in core bacteria abundance and an increase in subdominant bacteria, a decrease in sugar-splitting bacteria and an increase in proteolytic bacteria, a decrease in *Bifidobacteria* and an increase in certain proteobacteria, and also decrease in microbial diversity and decrease in the ratio of *Firmicutes* to *Bacteroidetes* [5]. Not only gut microbiota, but also the oral microflora, can be affected by and contribute to the aging of the body [6]. An adult produces more than 1000 mL of saliva per day, almost all of which enters the gastrointestinal tract. Hence oral microbes have a good chance of getting into the gastrointestinal tract with saliva or food, some of these oral microorganisms survive the competitive rejection of low pH gastric acid, hypoxia and the intestinal microorganisms, and colonize in the intestinal tract, becoming intestinal opportunistic pathogens [7–9]. For example, *Fusobacterium nucleatum*, a periodontal disease pathogen that has been identified as an important pro-inflammatory bacterium in human colorectal adenoma or cancer, can adhere to and invade intestinal epithelial cells through toxic factors such as FadA, Fap2, and FomA, promoting the proliferation of colon cancer cells and increasing the risk of poor prognosis [10–13]. Many resident oral bacteria have been detected in appendectomy samples from patients with acute appendicitis, such as *Gemella*, *Parvimonas,* and *Fusobacterium* [14, 15]. In addition, bad oral hygiene habits can change the oral mucosa microbiota, thus causing intestinal microbiota imbalance [16]. The deterioration of the oral environment can also lead to a deterioration in the overall health status of the body. Periodontitis is a major inflammatory disease of the oral mucosa, epidemiologically associated with other chronically inflammatory-driven conditions, including cardiovascular, metabolic, neurodegenerative, autoimmune diseases, and cancer [17]. There have been numerous reports about aging and aging related diseases and gut microbiota, but the relationship between oral microbiota and aging is relatively scantly reported. By summarizing the literature, this review focuses on the relationship of oral microbiota with aging and aging related diseases.

## Oral microbiome

Oral cavity is the beginning of digestive tract, the anterior interface fissure communicates with the external environment, intersecting through the pharynx gorge to the pharynx, respiratory system and digestive system. It is an important place for the transfer and exchange of internal and external environmental substances, as well as the first line of defense against pathogens and toxic substances [18]. In addition to its own tissues and secretions (such as teeth, mucosa, tongue, and saliva), human oral cavity is also an important microbial habitat colonized by microorganisms including bacteria, viruses, and microeukaryotes, which are collectively referred to as the oral microbiome [19, 20]. The configuration of the oral microbiome undergoes continuous modulation over the course of an individual’s lifespan, influenced by a myriad of elements encompassing genetic attributes of the host, maternal transfer, alongside environmental constituents like dietary patterns, oral cleanliness routines, pharmaceutical agents, and systemic determinants [21].

### Bacteria

**Figure 1.**
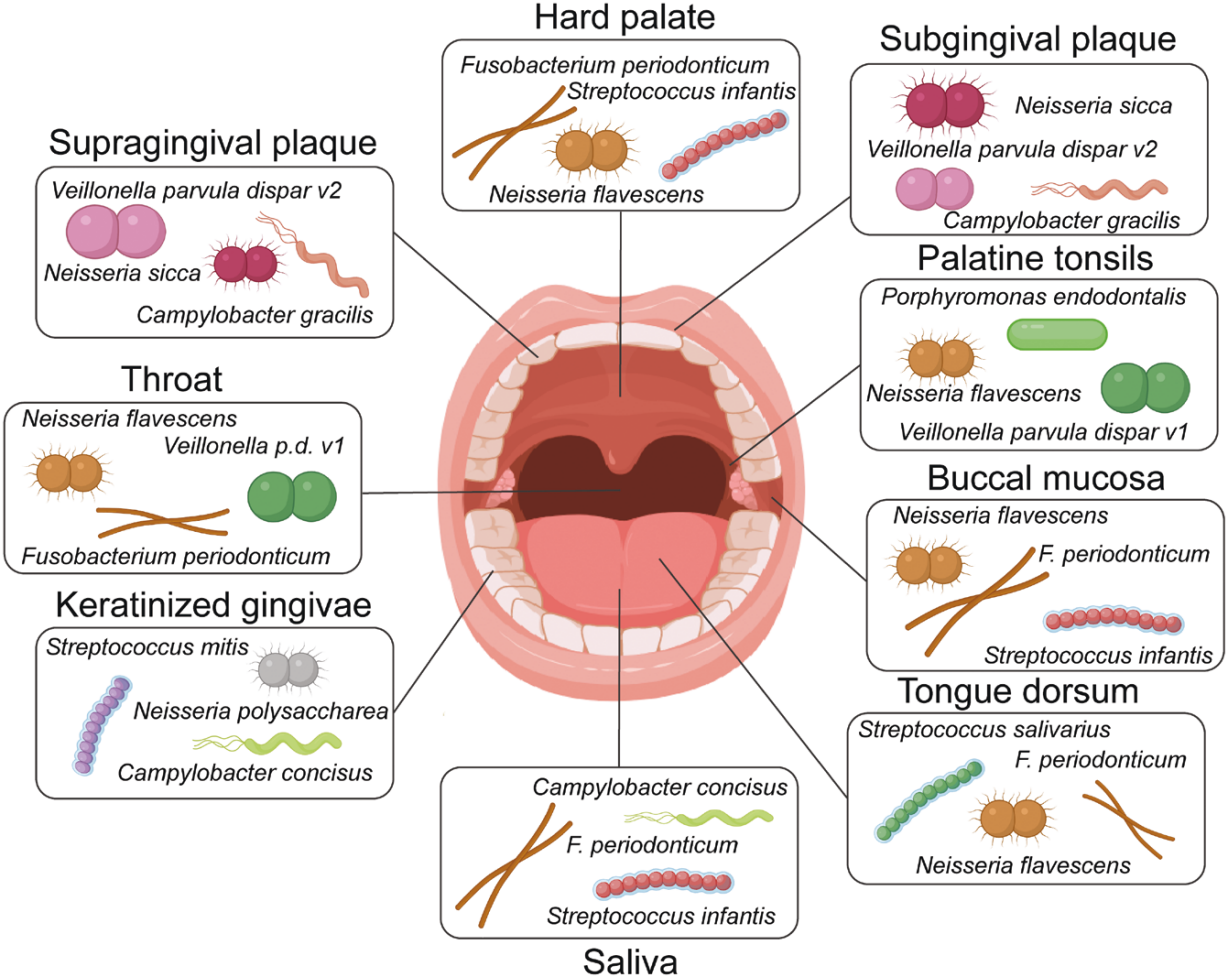
**The distribution of oral microbiome.**The oral cavity comprises a diverse range of microenvironments, making it a highly complex microbial habitat. Microbes colonize the hard surfaces of teeth and the soft tissues of the oral mucosa. Structures including hard palate, subgingival, supragingival, palatine tonsils, throat, buccal mucosa, keratinized gingivae, tongue dorsum, and saliva provide an ideal growth environment for microorganisms. The oral cavity maintains a stable temperature, averaging around 37°C, which offers bacteria a consistent habitat. Saliva maintains a stable pH range of 6.5–7, which is favorable for most bacteria, providing them with moisture and serving as a medium for nutrient transport. Microbial communities in different parts of the oral cavity exhibit distinct compositions and characteristics, the figure partially depicts the characteristics of oral microbiota, providing a simplified representation of oral microbial communities.

Currently, bacteria are the most studied group of oral microbiome compared fungi, protozoa, viruses and phages. Bacteria in different parts of oral cavity have different compositions and characteristics (Fig. 1). The plaque microbiota on the tooth surface is mainly aerobic bacteria, and subgingival plaque microflora is mainly facultative anaerobes and anaerobes [22]. Salivary microbiota shows relatively long-term stability, containing microbiota components of other sites, which can reflect oral microbiota composition to a certain extent [2, 23–25]. Human oral microbiome database summarizes up to 700 species currently found in the human oral cavity. In the database, only about 57% of the bacteria could be grown and named *in vitro*, while another 13% could be grown but not named, and about 30% could not be grown or named *in vitro* [26]. There is still a long way to go to fully understand the oral microbiota.

The oral cavity is one of the most diverse microbial habitats in the human body. Population studies showed that, comparing with other microbiomes in other parts of the human body, the bacteria generally show higher α diversity (abundance and evenness of species within samples) within subjects, and relatively lower β diversity (heterogeneity of species composition between samples) [23]. Also, there is individual heterogeneity in the degree of inflammatory response that the bacteria participate in and elicits [27]. Interestingly, even though there are some differences in bacterial composition between individuals at the same site, bacterial metabolism is relatively consistent [23]. This indicates that the basic conditions provided by specific local environment, such as oxygen concentration and nutrient composition, have a certain selective effect on the bacterial composition, and different bacteria in the microbiota can maintain the balance of metabolism through interactions.

The most abundant bacteria in the oral cavity are not exactly the same among individuals [28]. In general, *Streptococcus* is the most abundant genus in the oral cavity of healthy people, followed by *Haemophilus* on buccal mucosa, *Actinomyces* on gingival plaque, and *Prevotella* on subgingival plaque [28]. Bacterial composition in the oral cavity is influenced by disease status, heredity, diet, and living environment [21]. Poole et al. found that the copy number of salivary amylase gene significantly affected oral bacterial composition under the same diet conditions. Especially, high copy number of the human salivary amylase gene was associated with increased pathogenic bacteria such as *Porphyromonas*, while gut bacteria of people with high copy number of the human salivary amylase gene have increased abundance of resistant starch-degrading microbes, produced higher levels of short-chain fatty acids, and drive adiposity when transferred to germ-free mice [29]. Gomez et al. found that oral bacterial communities were more similar between twins, but this similarity decreased with age and sugar intake, and that it was environmental rather than genetic factors that affected tooth decay [30]. Shaw et al. found that, controlling for genetic factors, family members living in the same environment had more similar oral bacterial communities, and in general, environmental factors have more influence on oral bacterial composition than genetic factors [31].

### Microeukaryotes

In addition to bacteria, the oral microbiota also includes microeukaryotes (fungi and protozoa) as well as viruses (to be discussed later). Ghannoum et al. studied fungi in the oral cavities of 20 healthy adults, marking the first investigation of the “core mycobiome” in healthy individuals [32]. When oral infections occur, leading to microbial dysbiosis, fungal infections may be involved. Oral fungal infections are caused by various fungi, with common pathogens including *Candida*, *Aspergillus*, *Mucor*, *Histoplasma*, and *Blastomyces*, etc. These pathogens exhibit varying distribution patterns in the oral cavity, exerting different degrees of impact on patient health. *Candida* is one of the most common fungi implicated in oral fungal infections. Vila et al. pointed out that *Candida albicans* is the primary pathogen, responsible for approximately 95% of all cases of oral candidiasis. *Candida* can proliferate on oral mucosa, tongue, gums, forming white plaques accompanied by symptoms such as redness and pain. Oral infections with *Aspergillus* are less common, but when they occur, they often manifest as chronic inflammation or granulomas [33]. Sugui et al. found that among pathogenic *Aspergillus* species, *Aspergillus fumigatus* is commonly implicated [34]. *Mucor circinelloides*, typically found on human skin and mucous membranes, is less frequently associated with oral infections, likely due to its limited distribution on mucosal surfaces within the oral cavity [35]. *Histoplasma*, an intracellular parasitic fungus, primarily inhabits soil and decaying organic matter. It enters the human body through inhalation of airborne spores, leading to infection. Infected individuals may present with oral mucosal ulcers and granulomatous lesions. Folk et al. discovered that upon inhalation, the mycelial form of *Histoplasma* rapidly converts to its yeast form within the host [36]. *Blastomyces*, the etiological agent of chronic suppurative granulomatous diseases, exhibits intracellular parasitism. It enters the human body through minor skin or mucosal injuries, initiating infection. Cases of *Blastomycosis* are primarily reported in African and Middle Eastern countries, with Schwartz et al. finding that *Blastomyces* primarily causes human skin diseases, followed by pulmonary and osteoarticular diseases, with limited reports of its involvement in oral diseases [37].

Except fungi, there is another type of tiny single-celled organism in the oral microbiota, commonly known as oral protozoa. The diversity of protozoa in the oral cavity is limited, primarily including *Trichomonas tenax* and *Entamoeba gingivalis*. *Trichomonas tenax* parasitize within periodontal pockets and tonsillar crypts, often coexisting with dental abscesses [38]. Given that *Trichomonads* feed on bacteria and thrive in microaerophilic environments, the oral environment is conducive to the survival of *Trichomonas tenax* [39]. Some studies suggest that oral trichomonads are associated with the onset of periodontal diseases [40]. *E. gingivalis* is the only species of *Entamoeba* that colonizes the human oral cavity and is also associated with periodontal diseases [41]. Although the abundance of oral protozoa in the oral microbiota is relatively low, they are indispensable components of the oral microecosystem. Therefore, future research on oral protozoa is necessary as it can help elucidate the relationship between the human physiological system and the oral microenvironment.

### Viruses

As early as the 1990s, herpes simplex viruses (HSV) were recognized as one of the pathogenic factors in oral diseases. Studies suggest a possible association between herpes virus infection and the clinical manifestations of aggressive periodontitis [42]. The detection rate and abundance of herpes viruses vary with age, geographical location, and ethnicity. Reports from different countries confirm the high prevalence of herpes viruses in various oral diseases [43]. Slots summarized the results of over 20 studies worldwide and found the average infection rates of herpes simplex virus type 1 (HSV-1), Epstein-Barr virus (EBV), and human cytomegalovirus (HCMV) in chronic periodontitis patients to be 26%, 46%, and 52%, respectively; whereas in healthy populations, they were as low as 0%, 8%, and 8%, respectively [44]. In recent years, numerous studies have found a correlation between the detection rates of herpes viruses in periodontitis patients, mainly chronic and aggressive periodontitis, and the number of periodontal pathogens. Among these pathogens, the correlations to EBV, human cytomegalovirus, *Porphyromonas gingivalis*, and *Tannerella forsythia* are particularly high [45, 46]. It is speculated that herpes viruses and periodontal pathogens interact in periodontitis, with mechanisms including the adhesion and colonization of periodontal pathogens, activation of latent herpes viruses, and host immune suppression against periodontal pathogens.

Human immunodeficiency virus (HIV) was also among the earliest viruses recognized to impact the oral microbiota. HIV-infected individuals are susceptible to various oral microorganisms, leading to oral diseases. Opportunistic oral infections are correlated with the level of immune suppression and are considered indicators of disease progression and deterioration [47]. HIV infection and the reduction of related CD4^+^ T-cell counts disrupt the balance between host immunity and indigenous microbial communities, resulting in alterations in microbial composition and diversity. These changes may further affect oral tissue health, local immunity, and increase susceptibility to pathogenic microorganisms. Studies have found an increased diversity of oral microbiota in individuals infected with HIV, with some microbial species not found in healthy populations [48]. Mukherjee et al. analyzed oral rinse samples from 12 HIV-infected and HIV-uninfected individuals, discovering core oral bacteria present in both groups, with 13 genus species commonly found in both groups, indicating similar oral bacterial compositions between the two groups [49]. However, *Capnocytophaga* was exclusively present in the HIV-infected group, while *Aggregatibacter* was only present in the HIV-uninfected group. Conversely, core oral fungi differed between the two groups, with *Candida*, *Epicoccum*, and *Alternaria* predominant in the HIV-infected group, whereas *Candida*, *Pichia*, and *Fusarium* predominated in the HIV-uninfected group.

In addition to HSV and HIV, there is a large and diverse population of viruses present in the oral cavity, known as bacteriophages [50, 51]. Many bacteriophages play important roles in shaping the ecology of oral microbial communities. Bacteriophages can alter genetic structures through lysogenic conversion, providing new functions to host bacteria, offering selective advantages for host bacteria to cope with antibiotics or other types of interferons, and assisting host bacteria in breaking through mucosal barriers. Most bacteriophages in the human oral cavity belong to the families of *Siphoviridae* (mostly lytic bacteriophages with a moderate host range), *Myoviruses* (primarily lytic bacteriophages with a broad host range), and *Podoviruses* (mostly lytic bacteriophages with a narrow host range) [52, 53]. The relationship between the abundance of bacteriophages and their host bacteria has been a research hotspot, with most studies suggesting an inverse relationship between the abundance of host bacteria and bacteriophage abundance [54–56]. This is speculated to be related to the lytic and lysogenic cycles of bacteriophages. However, some studies have found that the relative abundance of *Streptococcal* bacteriophages correlates with changes in the relative abundance of their host bacteria, suggesting both antagonistic and symbiotic relationships between bacteria and bacteriophages in the oral cavity. Ly et al. compared the quantity and diversity of bacteriophages in saliva and plaque between periodontitis patients and periodontally healthy individuals, finding significant differences in the quantity and types of bacteriophages within plaque between the two groups, which correlated with periodontal status [57]. Meanwhile, the bacteriophage structure within periodontally healthy individuals remained relatively stable, suggesting a close association between bacteriophages and the pathogenesis of periodontitis. Due to their identity as bacterial viruses and their high specificity for certain types of bacteria, bacteriophages can be used to target specific species of host bacteria in specific environments, thereby influencing local microbiota and altering the course of disease.

In summary, the oral cavity harbors a large number of microorganisms, predominantly bacteria, with a diverse array of species. Bacteria,microeukaryotes, and viruses in the oral cavity interact with each other, and combined with the presence of multiple unique and complex anatomical structures such as mucosal soft tissues, teeth, and the special fluidic saliva, create various intricate microenvironments. Imbalance of oral microbiota can lead to the occurrence of local oral diseases such as dental caries, periodontal diseases, and oral cancer [58]. Moreover, it can also trigger systemic diseases through various pathways including direct transmission, hematogenous infection, and immune activation, such as cardiovascular diseases [59], digestive system diseases [60], and autoimmune diseases [61]. Aging is a significant risk factor for oral microbiota imbalance. With advancing age, physiological functions decline, immunity decreases, the barrier function of the oral mucosa weakens, saliva secretion decreases, and oral self-cleaning ability diminishes, leading to dysbiosis in the oral microenvironment and proliferation of pathogenic microorganisms. Therefore, studying the microbial ecology characteristics of the oral microbiota (especially the predominant oral bacteria) and risk factors is of great significance for a deeper understanding of the mechanisms underlying disease occurrence and ameliorate the effects of aging.

## Oral microbiota may influence gut microbiota

Saliva produced by human mouth constantly flows through esophagus into digestive tract, except during sleep [62–64]. Thus, saliva and food may carry microbes that colonize oral cavity into digestive tract. Oral microbiota colonization is minimal in a healthy intestinal tract. The colonization of indigenous gut microbiota in a healthy intestinal environment is considered a primary barrier to prevent the ectopic colonization of oral bacteria. However, the colonization of oral microbiota increases when there is an imbalance in gut microbiota [65].

In many diseases such as acquired immune deficiency syndrome (AIDS), cirrhosis, and inflammatory bowel disease (IBD), changes in the gut microbiota of patients can be traced back to oral-origin microbiota [7–9]. Studies have found ectopic colonization of oral bacteria in the gut microbiota of patients with cirrhosis [8]. In untreated patients with inflammatory bowel disease, specifically Crohn’s disease, two oral-origin microbes, *Veillonella* and *Fusobacterium*, are found in the gut microbiota and are closely associated with disease progression [9]. Recently, mice experiments have reported that oral bacteria can colonize the intestine, leading to activation of the intestinal immune system and chronic inflammation [66]. For example, Atarashi et al. transplanted Crohn’s disease saliva sample into germ-free mice by gavage and found that composition of Th1 cells in lamina propria significantly increased, and the dominant microbial species in the feces of mice were minor components of the salivary microbiota, suggesting a small part of oral microbiota species can indeed colonize the gut [67]. Feeding mice the oral bacteria *Klebsiella pneumoniae* 2H7 (Kp-2H7) increases the number of Th1 cells and activate TLR4 on dendritic cells in the intestine, prompting intestinal epithelial cells to secrete interleukin (IL)-8, further exacerbating inflammation [68, 69].

In short, oral microbiota can impact gut microbiota by migrating through saliva into the digestive tract. This colonization increases under gut microbiota imbalance. Diseases like AIDS, cirrhosis, and inflammatory bowel disease are linked to changes in gut microbiota originating from the mouth. Recent mouse studies show oral bacteria can colonize the intestine, leading to chronic inflammation. This underscores the connection between oral and gut microbiota and their influence on health.

## Relationship between oral microbiota and systemic diseases

**Figure 2.**
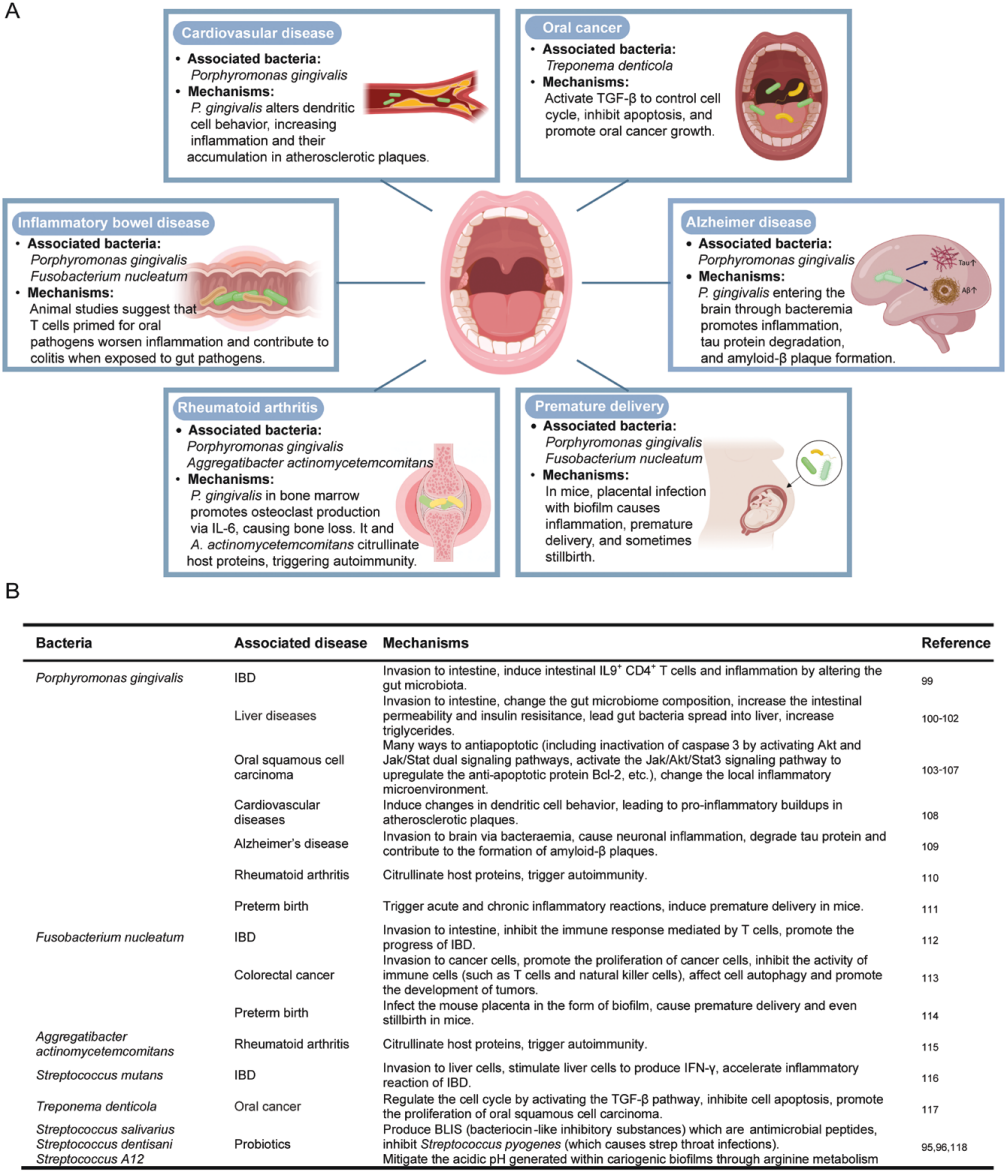
**The association between oral microbiota and human diseases.**(A) A summary of oral microbiome associated with human diseases. Numerous investigations have linked specific oral bacterial species and functional attributes to systemic diseases. The accompanying illustration underscores various such connections, offering detailed insights into the mechanisms involved. (B) Summary table of oral probiotics and the association of oral microbiota with human diseases.

As early as 1891, William Miller, regarded as the father of oral microbiology, proposed the theory of oral focal infection, pointing out that oral microbial infection can enter other parts of the body, whether adjacent or distant, leading to oral diseases as well as various systemic diseases [70]. In addition, proponents of the theory argue that plaque and its metabolites can enter the bloodstream, causing a variety of systemic or degenerative changes. Therefore, it is not only popular in dentistry but also in medicine to treat systemic diseases by pulling out bad teeth [71]. However, the theory of oral lesions has not received enough attention and theoretical support. In recent years, with the development of microbiome research, the relationship between oral microbiome and a variety of major chronic non-communicable diseases has been gradually confirmed (Fig. 2).

### Oral microbiome and oral diseases

For oral diseases, oral microbiota have been reported to cause various infectious oral diseases, including dental caries, periodontitis, pulp infections, alveolar osteitis, and tonsillitis. Among them, periodontitis is a chronic inflammatory disease caused by dental plaque, which may increase the risk of atherosclerosis, diabetes, rheumatoid arthritis, preterm birth, and pneumonia [72–76]. Studies have shown significant differences in oral microbial composition between patients with periodontitis and healthy individuals [77, 78]. The majority of periodontal disease pathogens are Gram-negative anaerobic bacteria residing in the gingiva, with the most important three oral pathogens being *P. gingivalis*, *Tannerella forsythia*, and *Treponema denticola*, which constitute the so-called “red complex” [79]. As for oral cancer, it was found that there were specific microorganisms on the surface of oral cancer and inside the oral cancer tissues. Oral cancer stands as the most prevalent malignancy within the domain of head and neck tumors and ranks among the most frequently diagnosed cancers globally. The year 2020 witnessed an approximate influx of 380,000 fresh cases, contributing to a toll of around 180,000 fatalities attributed to this condition [80]. Mager et al. found that *Capnocytophaga gingivalis*, *Prevotella melaninogenica,* and *Streptococcus mitis* abundance were elevated in patients with oral squamous cell carcinoma and these three bacteria can be used as diagnostic markers to diagnose about 80% of oral squamous cell carcinoma [81]. Therefore, these three bacteria have potential values as diagnostic indicators of oral squamous cell carcinoma. The metatranscriptomics analysis of the oral cancer-associated microbiome, or oncobiome, revealed distinctive functionalities executed by these bacteria, encompassing activities related to metal ion transport and nitrous oxide reductase, as well as tryptophanase and protease functions [58].

### Oral microbiome and digestive system diseases

In terms of digestive system diseases, oral microbiota is closely associated with inflammatory bowel disease (IBD). The presence of oral microbiota, including *C. concisus* and *F. nucleatum*, was first observed in the fecal metagenome and intestinal biopsy samples of IBD patients in 2010 and 2011 [82, 83]. Subsequently, more oral microbiome have been identified to colonize the intestines of IBD patients, encompassing organisms such as *Haemophilus parainfluenzae*, *Veillonella parvula*, *K. pneumoniae*, *Porphyromonas gingivalis* among others [9, 84]. A distinct correlation exists between periodontitis and IBD, with reports indicating more severe periodontal inflammation in IBD patients [60]. Additionally, compromised oral hygiene can alter the oral microbial community, leading to dysbiosis of gut microbiota and contributing to the onset of inflammatory bowel diseases [16].

### Oral microbiome and cardiovascular disease

Cardiovascular disease refers to the lesions occurring in the heart and vascular circulation system, including coronary heart disease, endocarditis, myocardial infarction, etc., with high mortality. A large number of case studies and epidemiological investigations have shown that periodontitis is an important risk factor for the development of cardiovascular disease [60, 85, 86]. In patients with periodontitis, gum epithelium in periodontal pocket is prone to breakage, which helps bacteria enter the systemic circulatory system, leading to bacteremia, or ectopic colonization of other organs in the body. Patients with periodontitis have a 25% increase in the likelihood of developing atherosclerotic plaque formation in arteries [59]. *P. gingivalis*, which is closely related to the occurrence of periodontitis, can invade and colonize atherosclerotic plaques in patients and can be detected in almost all atherosclerotic plaques [87]. *P. gingivalis* not only expedites the development of atheromatous plaques but also triggers the occurrence of fatty streaks within rabbit aortas [88]. Conversely, apolipoprotein E-deficient mice subjected to intraoral exposure to oral *S. sanguinis* exhibit significantly elevated expression of pro-inflammatory cytokines and an augmented propensity for atherosclerotic plaque formation [89]. Bacteria associated with periodontal disease may destroy body’s immunity, stimulate cells to produce inflammatory factors such as TNF-α, IL-1β, and IL-6, causing related inflammation and vascular endothelial injury and promoting the formation of atherosclerotic plaque.

### Oral microbiome and rheumatoid arthritis

Rheumatoid arthritis (RA) is a chronic, systemic autoimmune disease characterized by synovial hyperplasia, synovitis, erosion of bone and cartilage, resulting in joint swelling, symmetrical pain, and stiffness, ultimately leading to joint deformity and restricted mobility, severely affecting quality of life [90]. Its occurrence and development represent a chronic and irreversible pathological process. Despite significant advances in immunology, genetics, cell biology, and biochemistry, the etiology and pathogenesis of RA remain incompletely understood [90]. It is generally believed to result from the interaction of host genetic factors and environmental factors leading to immune system dysregulation and disease onset. Globally, approximately 0.5%–1% of the population is affected by this disease, with higher prevalence rates among native American populations, particularly the Pima and Chippewa tribes, reaching up to 5.3% and 6.8% [91], while lower rates are observed in China and Japan, with rates around 0.2%–0.3% in China [92]. There is substantial evidence indicating that the pathogenesis of periodontitis and RA is very similar, and both diseases share many similarities in terms of genetic susceptibility, clinical phenotype, and conventional treatment strategies. The proportion of RA patients with periodontitis is very high, and similarly, patients with periodontitis have a higher prevalence of RA. These two diseases also exhibit many similarities in pathological manifestations, such as soft tissue damage, bone erosion, and secretion of inflammatory cytokines like IL-1, indicating a close relationship between periodontitis and RA [93]. A study found that periodontal disease-associated oral mucosal breaks result in oral microbiome that trigger innate and adaptive immune responses, which, when repeated over time in a susceptible host, likely contribute to the pathogenesis of RA [94]. In mouse models, IL-6 is important for *P. gingivalis-*induced accumulation of CD11b^+^c-fms^+^Ly6C^hi^ population from the bone marrow and periphery, and it suggests the invasion of *P. gingivalis* can disrupt the development of immune cells, promote osteoclastogenesis, and lead to bone loss [95]. Additionally, DNA of *P. gingivalis* and high titers of anti-*P. gingivalis* antibodies were detected in the serum and synovial fluid of RA patients [96].

### Oral microbiome and premature delivery

Intrauterine infection may lead to many complications during pregnancy and is one of the risk factors for premature delivery. Mendz et al. found through meta-analysis that among 761 preterm pregnant women, 349 had intrauterine infection with up to 87 pathogens, including respiratory tract pathogens and common oral microorganisms in addition to reproductive tract pathogens [97]. Compared with sites such as vagina, intestine, and respiratory tract, the composition of placental microbial community is most similar to that of oral microbiota, and maternal periodontitis may serve as a potential risk factor for adverse pregnancy outcomes [98]. In an animal model study, Fardini et al. identified a portion of the oral microbiota that can translocate to the murine placenta following hematogenous infection [99]. Oral bacteria such as *Capnocytophaga*, *Tannerella*, *Treponema*, *Peptostreptococcus* were also detected in amniotic fluid [99]. Evidence from animal studies validates that oral infection with *P. gingivalis* elicits a 2.5-fold elevation in maternal serum cytokine levels of tumor necrosis factor-alpha, a 2-fold increase in IL-17, a 2.5-fold increase in interferon gamma, a 2-fold rise in IL-6, and a 2-fold rise in IL-1β. Additionally, this infection amplifies the expression of toll-like receptor 2 and mediators associated with the Fas/Fas ligand pathway within placental tissues, subsequently resulting in the induction of preterm birth and low birth weight [63, 100].

### Oral microbiome and Alzheimer’s disease

Alzheimer’s disease is a degenerative disease of the nervous system. In a retrospective analysis, data from 262,349 participants within the Korean National Health Insurance Screening Cohort were examined [101]. When compared to individuals without periodontitis, those afflicted with chronic periodontitis exhibited an increased susceptibility to overall dementia (adjusted hazard ratio = 1.06; 95% confidence interval = 1.01–1.11) as well as Alzheimer’s disease (adjusted hazard ratio = 1.10; 95% confidence interval = 0.98–1.22) [102]. Furthermore, a recent longitudinal study reported that individuals enduring chronic periodontitis for a minimum of 10 years displayed a heightened risk of developing Alzheimer’s disease, along with a heightened prevalence of hyperlipidemia, depression, traumatic brain injury, and comorbid conditions compared to counterparts without periodontitis [103]. Earlier, Miklossy et al. suggested that oral microorganisms were closely related to the occurrence of Alzheimer’s disease [104]. This hypothesis was strongly supported by the detection of oral spirochetes and *P. gingivalis* lipopolysaccharides from brain tissue by molecular biological techniques [105]. Consistently, animal studies have demonstrated that repeated administrations of *P. gingivalis* or *P. gingivalis* lipopolysaccharide (LPS) have deleterious effects on spatial learning and memory of mice as assessed in behavioral paradigms like the Morris water maze [106–111]. This decrement in cognitive performance was observed exclusively in middle-aged mice (10 weeks or older), but not in young mice [107, 111]. Prolonged administration of *P. gingivalis* or *P. gingivalis* LPS elicited a systemic immune response characterized by heightened levels of pro-inflammatory cytokines such as IL-1β, IL-6, TNF-α, among others. This immune response aligns with general findings from periodontal animal models. Regarding Alzheimer’s disease-specific markers, Nie et al. found that intraperitoneal injection of *P. gingivalis* led to increased mRNA expression of AβPP770, an amyloid β protein precursor, in liver tissue [112]. Histologically, chronic administration of *P. gingivalis* or *P. gingivalis* LPS resulted in an augmentation of activated microglial cells and astrocytes within the cortex [113] and hippocampus [114, 115], serving as indicators of local inflammation. Levels of pro-inflammatory mediators, including IL-1β, IL-6, TNFα, and INF-γ, were found to be elevated at both mRNA and protein levels in comparison to control groups [106, 107, 109, 114–116]. After invading the brain, oral bacteria and the endotoxin they release may activate microglia cells, further leading to the release of pro-inflammatory cytokines such as TNF-α and IL-1, while brain tissue exposed to high concentrations of TNF-α and IL-1 for a long time may make it easier for bacteria or endotoxin to infiltrate the brain tissue and aggravate the clinical symptoms of the disease [117]. So oral microbiota may also be associated with nervous system diseases.

In summary, William Miller’s theory of oral focal infection, proposed in 1891, highlights the role of oral microbial infections in causing both oral and systemic diseases. This theory suggests that oral pathogens can disseminate to various parts of the body, leading to conditions such as periodontitis, cardiovascular disease, inflammatory bowel disease, intrauterine infections, and even neurodegenerative diseases like Alzheimer’s. Studies have revealed significant associations between oral microbiota and these diverse health conditions, emphasizing the importance of oral health to overall well-being. Therefore, understanding and managing oral microbiota could have significant implications for preventing and treating a range of systemic diseases.

## Oral microbiota and aging

**Figure 3.**
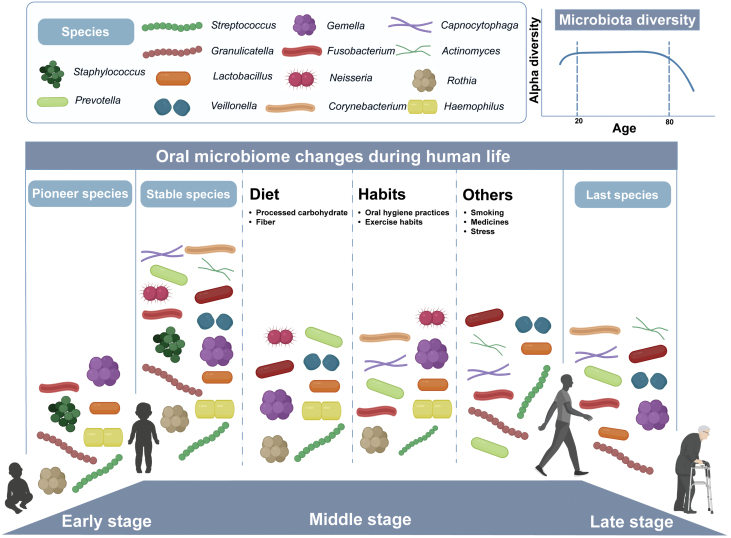
**The succession of oral microbial communities from birth to aging.**Early stage: Microbial colonization initiates shortly after birth through vertical transmission from the mother, dietary transmission, and interactions among infants and other humans. The diversity of microbes expands when primary teeth erupt, creating additional microbial habitats in the oral cavity. This eruption of primary teeth also leads to a shift away from the maternal oral microbiota [122, 123]. Middle stage: With the progression of age in children, the oral microbiotas tend to reach a more stable state [21, 124]. Throughout life, the oral microbiota remains subject to influence from many environmental factors. Environmental factors that impact the composition and functions of the oral microbiome encompass diet, stress, oral hygiene practices, medications, physical activity, and smoking [125, 126]. Late stage: There is relatively limited research on the changes in oral microbiota during the aging process. Studies have indicated a reduction in oral microbiome diversity in individuals older than 80 years old [127]. This figure provides a general schematic overview of the changes in oral microbiota and does not encompass all variations in oral microbial communities.

The oral microbiome of healthy individuals undergoes dynamic changes throughout development and aging, with specific alterations in composition across different age groups. During the deciduous dentition stage, *Proteobacteria*, including *Acinetobacter* and *Moraxella*, dominate, while *Bacteroidetes* become predominant in the mixed and permanent dentition stages, as age advances, there is a gradual increase in the abundance of *Prevotella*, *Veillonellaceae*, *Spirochetes*, and TM7 [118]. Xu et al. conducted a horizontal comparative study on oral microbial components across different ages, revealing that *Firmicutes* predominate in the adolescent stage, transitioning to *Proteobacteria* in adulthood, and with an increase in the abundance of *Actinobacteria* in older age [6]. Moreover, studies by Percival et al. and Preza et al. indicated significant changes in the oral microbiota composition with aging, including an increase in *Actinomyces* abundance in individuals aged 60 and above [119], and potentially higher bacterial diversity in elderly individuals without dental caries or periodontitis compared to younger counterparts [120]. Additionally, Liu et al. observed variations in microbial alpha diversity across different age groups, with the highest diversity observed at ages 11–15 and 18–20, and beta diversity increasing with age [121]. It indicates that oral microbiota composition is related to the development and aging of the body, and different age stages are marked by different dominant oral microbial species (Fig. 3).

Wolford first proposed in 1969 that immune dysfunction was highly associated with aging which is called immunosenescence [128]. In 2000, Franceschi suggested that innate immunity is activated with aging, leading to a low level of chronic inflammatory state, namely inflammatory aging (inflamm-aging) [129]. Oral microbiota imbalance can also cause inflammation, such as periodontitis, which is the most common oral inflammatory disease. In healthy conditions, oral microbiota maintains a dynamic balance with the local host environment. However, in individuals susceptible to frailty and improper oral hygiene maintenance, the balance is easily broken, and the number of pathogenic bacteria significantly increases. When chronic periodontitis occurs, subgingival microbiota changes from those with *Streptococcus* and *Prevotella* as the main populations to those with *Prevotella* and *Clostridium* as the main populations [130]. The microbiota developed from non-pathogenic structure to pathogenic structure, and microbiota metabolism also changed from anabolism to catabolism [130]. For example, *P. gingivalis*, a key pathogen causing periodontitis, contains many pathogenic factors such as *P. gingivalis* peptidylarginine deiminase (PPAD), capsule, pili, gingipain R(Rpg) and gingipain K(Kpg) [131]. These pathogenic factors show functional polymorphism in different strains, which can affect physiological characteristics and pathogenicity of bacteria. For example, Rpg secreted by the bacteria can degrade proteins into short peptides containing arginine at the carbon end, which are further modified by PPAD citrulinization, thus leading to the production of autoantibodies in human body and promoting the occurrence of rheumatoid arthritis [61]. Therefore, oral microbiota when causing inflammation in human body, perhaps also induces inflamm-aging. During aging, the prevalence of systemic diseases also increases. Among elderly individuals with poor health status, the oropharyngeal region may harbor a number of opportunistic *Enterobacteria*, *Candida* spp. yeasts (primarily *C. albicans*), and *Staphylococci*. Oral *Candidiasis* is a common bacterial infection in the elderly, and physiological changes in the oral mucosa, micronutrient deficiencies, malnutrition, and other factors facilitate the colonization of *C. albicans*. Oral diseases in the elderly population may trigger low-grade chronic systemic inflammation [132], posing a significant morbidity and mortality risk. For instance, dental procedures aimed at restoring oral health in older adults could paradoxically result in bacteremia and further augment systemic inflammation in elderly patients [133]. Regarding the relationship between oral microbiota and aging, there remains substantial work to be done to confirm and pinpoint the contribution of oral microbial communities to the aging process.

In summary, the oral microbiome undergoes dynamic changes during individual development and aging, with specific alterations in composition across different age groups. Aging is associated with immunosenescence and inflamm-aging, where oral microbiome imbalance, such as in periodontitis, may lead to chronic systemic inflammation. Oral hygiene is closely linked to health during aging, as poor oral health in elderly individuals predisposes them to opportunistic infections like oral candidiasis, potentially exacerbating systemic inflammation and increasing the risk of morbidity and mortality. Further research is needed to elucidate the precise role of the oral microbiome in the aging process.

## The role of oral microbiota in disease prevention, therapy and prognosis

Dental caries refers to microbial destruction or necrosis of teeth. Traditional caries risk assessments include single bacterial parameter (such as *S. mutans* and *Lactobacillus* colony count), saliva biochemical parameters (saliva flow rate, pH, and buffer capacity testing), caries disease history, individual oral hygiene, brushing habits, eating habits, and social and economic conditions, etc. [133, 134]. Teng et al. found through high-throughput sequencing that the changes in oral microbiota preceded the clinical symptoms of dental caries, and based on this, the “microbial indicators of caries (MiC)” was constructed [135]. MiC does not depend on the subjective judgment of dentists or patients, thus can be objective in clinical dental decay before the disease onset (i.e. sub-health state), with 81% accuracy to predict the occurrence of early childhood caries, which can effectively help in clinical intervention and early diagnosis.

In addition, the oral microbiome of patients with rheumatoid arthritis is significantly disordered compared with that of healthy people, and this ecological imbalance can be recovered by the treatment of rheumatoid arthritis, and the degree of recovery is closely related to the response of patients to treatment. According to the metagenomic association analysis of oral and intestinal microbiota, the diagnostic accuracy of a classification diagnosis model is nearly 100% to differentiate healthy people and patients with rheumatoid arthritis.

Another endeavor aiming to utilize oral microbial abundances as biomarkers has exhibited initial promise in the early identification of colorectal (CRC) lesions [136]. The conventional non-invasive screening methods for detecting CRC, such as the fecal immune test (FIT) and the fecal occult blood test (FOBT), demonstrate limited sensitivity in detecting early-stage lesions. In this context, the researchers demonstrated that a classification model derived from oral microbiome samples exhibited elevated specificity and enhanced sensitivity compared to conventional FIT-based assays in discriminating CRC and polyp samples from healthy controls. Notably, a further improvement in sensitivity was achieved by integrating fecal microbiome samples into the analysis.

Apart from serving as a biomarker, the oral microbiome can function as both a therapeutic tool and a focal point for disease management. As the entrance to the digestive system, oral cavity is the transportation hub connecting the human body. The oral microbiome is fine-tuned by human health and disease states, and quickly adapt and magnify the body’s preclinical symptoms. In the prevention and clinical treatment of digestive tract tumors, eliminating oral *Helicobacter pylori* can assist in the prevention of gastric tumors. At present, the most commonly used treatment method to eradicate *H. pylori* in the stomach is conventional triple therapy, that is, proton pump inhibitor combined with two antibiotics, although the recurrence rate is high. Using drugs combined with periodontal therapy to control *H. pylori* in oral cavity can reduce the recurrence rate of *H. pylori* infection in the stomach. In chronic periodontitis patients with gastric *H. pylori* infection, the eradication rate of *H. pylori* is improved after systemic drug therapy combined with periodontal therapy [137, 138]. Bouziane et al. performed a meta-analysis on the effect of periodontal therapy on eradication rate of *H. pylori* gastric infection and found that periodontal therapy could reduce the recurrence rate of *H. pylori* gastric infection [139]. The above research indicates that *H. pylori* infection in the stomach can be controlled by controlling *H. pylori* in the oral cavity, which is of great significance for the early prevention of gastric tumors. Therefore, it might be pivotal to control the infection of certain oral microbiota for the early prevention of digestive tract tumors.

Probiotics that are readily available in the market, containing viable strains of bacterial genera such as *Bifidobacterium*, *Lactobacillus*, and *Streptococcus*, have been demonstrated to enhance the alpha diversity within the oral microbiome, albeit without causing extensive or persistent modifications to its composition [140]. Proposed mechanisms also underlie the potential beneficial impact of other probiotics in countering the progression of periodontal disease and caries. Notably, strains of *Streptococcus salivarius* have exhibited the capacity to mitigate inflammatory responses and stimulate immune pathways, including type I and II interferon responses [141]. Other potential probiotics for this purpose encompass *S. dentisani* [142] and *Streptococcus A12* [143], both of which can mitigate the acidic pH generated within cariogenic biofilms through arginine metabolism (Fig. 2B). In addition, in Alzheimer’s disease, inhibitors targeting the gingipains proteases secreted by *P. gingivalis* have demonstrated the capability to mitigate infection by this species in the brain, along with curtailing amyloid-β production and neuroinflammation [144].

In short, the oral microbiome plays a significant role in various aspects of health and disease. It has been suggested that the oral microbial community can adapt to the recovery of body diseases, and is highly sensitive to the occurrence, development and prognosis of systemic diseases [145]. Till now, it has been utilized for caries risk assessment, with microbial indicators showing promise for early detection of dental caries. Moreover, alterations in the oral microbiome have been linked to conditions like rheumatoid arthritis and colorectal lesions, offering potential diagnostic and therapeutic avenues. Additionally, controlling certain oral microbiota, such as *H. pylori*, may contribute to the prevention of digestive tract tumors. Probiotics have emerged as a potential intervention to modulate the oral microbiome and mitigate conditions like periodontal disease and caries. Overall, understanding and harnessing the oral microbiome holds promise for disease management and prevention.

## Conclusions and future perspectives

In summary, the diversity and polymorphism of oral microbiota are factors affecting the systemic or chronic inflammation and the development, therapy, and prognosis of diseases. Therefore, oral microbiota is of pivotal importance to human body. As chronic inflammation is a key feature of driving the aging process, it would be unsurprising if the deterioration of oral microbiota health is also a driving factor in aging. However, so far there is hardly any study to specifically analyze oral microbiota in aging, despite there have been an enormous literature on gut microbiota in aging. With the development of high-throughput deep sequencing and quantitative mass spectrometry technologies and easy access to the oral microbiome samples, metagenomics and metabolomics of the oral microbiota in human aging will soon flourish. These will undoubtedly clarify the intrinsic and extrinsic relationships of oral microbiota with aging and aging-related diseases. At the same time, the traditional bacteriological research is particularly important for understanding bacterial gene function, pathogenic mechanism, bacterial interaction, etc. With the in-depth study, oral microbiota is expected to provide new ideas, approaches and practical applications to clinical interventions and treatment of aging and diseases in the near future.
